# Factors associated with posttraumatic growth in patients with severe burns by treatment phase

**DOI:** 10.1002/nop2.582

**Published:** 2020-08-03

**Authors:** Sun‐mi Hwang, Eun Ju Lim

**Affiliations:** ^1^ Hallym University Hangang Sacred Heart Hospital Seoul Republic of Korea; ^2^ Red Cross College of Nursing Chung‐Ang University Seoul Republic of Korea

**Keywords:** burns, depression, patients, posttraumatic growth, rehabilitation, social support

## Abstract

**Aim:**

This study aimed to identify differences in the level of depressive symptoms, social support and posttraumatic growth among patients with severe burns by treatment phase and the factors associated with posttraumatic growth in the acute and rehabilitation phases.

**Design:**

A cross‐sectional descriptive design.

**Methods:**

The factors associated with posttraumatic growth in 179 patients with severe burns were assessed using regression analysis.

**Results:**

Compared with those in the acute phase (mild depression), those in the rehabilitation phase showed moderate depression and lower positive self‐perception, resulting in a significant difference in the means. Social support was significantly correlated with posttraumatic growth and explained 14.9% of its variance in the acute phase. Depressive symptoms and social support were significantly associated with posttraumatic growth and explained 28.2% of its variance in the rehabilitation phase. Therefore, the need for counselling support and intervention for patients with severe burns is evident.

## 1 INTRODUCTION

Burns usually result from sudden accidents and the greater the severity, the higher the treatment costs and duration (Kil & Lee, 2019). Severe burns cause hypovolemic shock and wound sepsis (Brusselaers et al., 2010). Additionally, severe burns cause chronic low immunity and malnutrition and increase the rates of long‐term mortality as well as early death (Brusselaers et al., 2010). Nevertheless, survival rates have gradually improved owing to a better understanding of the pathophysiology of patients with severe burns, improved airway management of those with inhalation injuries, skin graft surgery and effective infection management (Alvarado, Chung, Cancio, & Wolf, 2009; Smolle et al., 2017). Recently, there has been a rise in interest in the psychosocial aspects of severe burns to improve recovery and approaches involving therapeutic teams of skilled medical personnel in various fields are being emphasized (Al‐Mousawi, Suman, & Herndon, 2012).

In patients with burn injuries, the acute phase starts after the emergency phase – the first 48 hr when treating the wound are of the utmost priority. In patients with higher than second‐degree burns, the onset of the acute phase can take several weeks, sometimes months and the appropriate treatments include continuous dressing, postural adjustments to prevent contractures and skin grafts (Lewis, Dirksen, Heitkemper, Bucher, & Harding, 2014). Following the acute phase, the rehabilitation phase entails the provision of care for small residual wounds and patients continue receiving treatments for unstable skin conditions, pruritus and physiotherapy; reconstructive surgeries may also be performed for those with severe burns (Lewis et al., 2014). Patients with burn injuries need management and treatment suitable for each phase considering the order of priority (Zachariah et al., 2012). As the burn injury becomes more severe, there may be repeated surgeries and attempts at rehabilitation, resulting in changes in patients’ psychological state and interpersonal relationships and even social maladjustment (Weon & Han, 2018).

## 2 BACKGROUND

Depression, which occurs in 52% of patients with burn injuries, is the most common symptom aside from physical problems (Hudson, Al Youha, Samargandi, & Paletz, 2017). Patients with burn injuries experience physical changes and the subsequent mental and psychological shocks can develop into depressive symptoms (Lee, 2017). Generally, patients with burn injuries focus on the recovery of physical and physiological functions when they first become hospitalized and then experience psychological pain from concerns regarding the success of the reconstructive surgeries, pain from the surgeries and anxiety about the after effects post the physical recovery phase (Lee, Ahn, Yoo, & Park, 1998; Lee, 2017). Additionally, the seriousness of the depressive symptoms is positively correlated with the severity of pain from the burn (Son, 2005) and the area of the burn (Al Ghriwati et al., 2017). Therefore, the early discovery and treatment of depressive symptoms are important in the context of the psychosocial health of such patients (Carmean et al., 2018).

Patients with burn injuries face difficulties in post‐discharge socialization and experience social isolation owing to the limitations of bodily functions in their daily lives (Weon & Han, 2018). In such a scenario, social support can serve to improve health management and social adaptation (Weon & Han, 2018). Through the process of socialization, patients with burns continue to interact with people in the outside world with whom they have significant relationships, including family members, medical staff and fellow patients with burns (Kil & Lee, 2019).

Contrary to the notion that patients with burns are restricted by their physical injuries and the complex problems that follow, including psychological changes and social adaptation, the burn incident may, in fact, serve as a turning point in their lives (Weon & Han, 2018). Psychological growth stemming from the desire to find meaning in the incident and subsequent injury can be observed in the cognitive phase (Calhoun & Tedeschi, 1999). Posttraumatic growth during psychological recovery may lead to a psychological state that is more positive than that before the burn incident (Martin et al., 2017).

Most studies on posttraumatic growth have focused on cancer; a study on patients with breast cancer reported that those with higher posttraumatic growth showed lower depressive symptoms (Carver & Antoni, 2004), while another study on cancer survivors showed that posttraumatic growth positively influenced quality of life and lowered depression (Morrill, 2011). However, only a few studies have examined the factors associated with posttraumatic growth by treatment phase. Therefore, this study sought to identify correlations among the major factors in patients with severe burns, so as to provide basic data for the development of nursing interventions focusing on psychosocial aspects.

Specifically, the study addressed the following research questions:

- Are there any differences in the level of depressive symptoms, social support and posttraumatic growth in patients with severe burns by treatment phase?
- Is there a correlation between depressive symptoms, social support and posttraumatic growth in patients with severe burns by treatment phase?
- Do depressive symptoms and social support of patients with severe burns by treatment phase affect posttraumatic growth?

## 3 METHODS

### 3.1 Research design

This study employed a cross‐sectional descriptive design.

### 3.2 Classification criteria

In this study, only adult burn patients were recruited; their severity levels were classified based on the extent and depth of injuries. The range of burns was assessed using Pulaski and Tennison's rule of nines, which is the ratio of burns on the body to the total body surface area. Patients with severe burns were selected according to the criteria of the Korean Society of Plastic and Reconstructive Surgeons (Korean Society of Plastic & Reconstructive Surgeons, 2019): second‐degree burns on 25% or more of the body surface area or third‐degree burns on the face, neck and joints. The categorization of treatment phases was based on the fact that the mean first hospitalization from third‐degree burns from 2013–2017 was 53 days (Health Insurance Review & Assessment Service, 2017). Aside from those who were in the emergency treatment phase, those in the acute phase were determined as patients hospitalized within approximately 2 months of the emergency phase. An average of 64 days had passed since the burn incident for those in the acute phase. Those in the rehabilitation phase visited as outpatients after being discharged were hospitalized in the rehabilitation department for physiotherapy, were receiving other clinical treatments, or were hospitalized in the plastic surgery department for reconstructive surgeries. On average, 685 days had passed since the burn incident for those in the rehabilitation phase. Those who could not communicate nor had mental disabilities were excluded. Furthermore, for patients with physical limitations due to burns, including visual impairment or wounds on the hands, one of the researchers read the survey questions aloud and marked their responses.

### 3.3 Data collection

Data were collected from outpatients and inpatients at H Burns Hospital in Seoul, Korea. This institution is a rehabilitation‐certificated hospital as well as a burn‐specialized hospital designated by the Ministry of Health and Welfare and provides surgical treatment to more than 2000 patients with severe burns in the acute phase every year. Data were collected from 11 March–3 May 2019. The study proposal was reported to the nursing department; the surveys were then distributed after obtaining board approval for data collection. The department heads of the burn units were given sufficient explanations regarding the purpose and process of the survey, along with a list of the patients satisfying the criteria for severe burns from which the sample was selected after excluding those who met the exclusion criteria. It took approximately 10 min to finish the survey, and data were mostly collected in the hospital's outpatient clinic or counselling room. G*Power 3.1 was used to calculate the sample size needed for the regression analysis (Faul, Erdfelder, Buchner, & Lang, 2009). The minimum sample size was 68, based on a significance level (α) of 0.05, power (1‐β) of 0.80 and medium effect size of 0.15. Finally, 68 patients in the acute phase and 111 in the rehabilitation phase were enrolled. In total, 179 surveys were distributed, and the return rate was 100%.

### 3.4 Instruments

#### 3.4.1 General and burn‐related characteristics

The general characteristics included five items on age, gender, educational level, financial hardships and personality (extroverted, introverted). Burn‐related characteristics were assessed through four items: the degree of burn as judged by a physician (%), quantification of burn‐related pain on a numeric rating scale, number of burn areas (upper and lower limbs, face, body, anus, genitals, etc.) and difficulties in daily life owing to burns.

#### 3.4.2 Depressive symptoms

The Beck Depression Inventory II, a modification of Beck’s (1967) work undertaken by the Korea Psychology Corporation (2017), was used to measure depressive symptoms. This 21‐item instrument is rated on a four‐point Likert scale from 0–3. Total scores range from 0–63, with higher scores indicating more severe depressive symptoms. Scores 0–13 are considered normal, 14–19 indicate mild depression, 20–28 indicate moderate depression and 29–63 indicate severe depression. Cronbach's α during the development of the instrument was 0.85 and in this study was 0.94.

#### 3.4.3 Social support

This variable was measured using the Social Support Scale developed in the Korean language by Lee (1994). This scale was published as a book at the Behavioral Sciences Institute affiliated with Korea University (1998). Since then, many researchers have used this scale to conduct research on social support. Factor analysis was used to determine the construct validity of this scale and yielded the following domains and items: emotional support, consisting of 7 items on love, empathetic listening, intimacy, trust, interest, encouragement and understanding; evaluative support, consisting of six items on fair evaluation, respect for personality, praise, talent recognition, value increase and respect for opinion; information support, consisting of 6 items on problem solving, decision‐making, adaptation, advice, recommendation and guidance provided in a crisis situation; and material support, consisting of 6 items on money, direct indirect help, services, lending goods, time and work. Each item is scored on a five‐point Likert scale ranging from 1, which means “strongly disagree” – 5, which means “strongly agree,” resulting in a possible score range of 25 to 125. Higher scores indicate higher social support. In this study, Cronbach's α was 0.86 for emotional support, 0.87 for evaluation support, 0.71 for information support and 0.91 for material support. Cronbach's α at the time of development was 0.92 and in this study was 0.97.

#### 3.4.4 Posttraumatic growth

Tedeschi and Calhoun’s (1996) Posttraumatic Growth Inventory is a widely used tool and, in this study, we used the version translated and adapted to the South Korean context by Song, Lee, Park, and Kim (2009). It has four categories (changes in interpersonal relationships, changes in self‐perception, discovering new possibilities, increased spiritual and religious interest) with 16 items scored on a six‐point Likert scale from 0, signifying “I did not experience this change” – 5, signifying “I experienced this change to a very great degree.” Total scores range from 0–80, with higher scores indicative of more positive posttraumatic growth experiences. Cronbach's α in Tedeschi and Calhoun’s (1996) study, Song et al.’s (2009) study and the present study was 0.90, 0.91 and 0.92, respectively.

### 3.5 Ethics

This study was conducted after receiving approval from the institutional review board of the institution to which the author is affiliated. Respondents received explanations regarding the purpose and methods of the study and participated voluntarily after providing written informed consent. They were informed that there would be no disadvantages for not participating or withdrawing during the process and that the surveys would: (1) be numbered to maintain anonymity and (2) be used for the purpose of only this study and then discarded. The data were kept in a secure vault.

### 3.6 Data analysis

The collected data were analysed using SPSS Statistics 25.0 (SPSS Inc). Frequencies, percentages, means and standard deviations were used for the general and burn‐related characteristics and the chi‐square test and *t* test were used to verify the homogeneity of the two groups by treatment phase. Depressive symptoms, social support and posttraumatic growth in the two groups by treatment phase were analysed using independent sample *t* tests and correlations were analysed using Pearson's correlation coefficients. The factors associated with posttraumatic growth by treatment phase were identified with the input method of regression analysis.

## 4 RESULTS

### 4.1 Sociodemographic and burn‐related characteristics by treatment phase

There were 179 participants and their mean age was 45.8 (*SD* 12.89) years. Among them, 38% were in the acute phase (*N* = 68) and 62% were in the rehabilitation phase (*N* = 111). There was homogeneity in age, gender, educational level, financial hardships, personality, degree of burns, pain from the burn, number of burn areas and difficulties in daily life for each treatment phase (Table 1).

**Table 1:** Sociodemographic and Burn‐related Characteristics by Treatment Phase

Variables	Total (*N* = 179, 100%)	Acute phase (*N* = 68, 38%)	Rehabilitation phase (*N* = 111, 62%)	t/χ^2^ (*P*)
	*N* (%)	*N* (%)	*N* (%)	
**Age (years) Mean ± *SD***†	45.8 ± 12.89	42.7 ± 13.66	47.6 ± 12.08	−2.530 (0.102)
**Gender**				0.445 (0.505)
Male	139 (77.7)	51 (75.0)	88 (79.3)	
Female	40 (22.3)	17 (25.0)	23 (20.7)	
**Duration of education (years)**				3.851 (0.146)
≤ 9	17 (9.5)	10 (14.7)	7 (6.4)	
10–12	84 (46.9)	32 (47.1)	52 (46.8)	
≥ 13	78 (43.6)	26 (38.2)	52 (46.8)	
**Financial difficulty**				6.473 (0.226)
None	32 (17.9)	18 (26.5)	14 (12.7)	
A little	79 (44.1)	24 (35.3)	55 (49.5)	
A lot	68 (38.0)	26 (38.2)	42 (37.8)	
**Personality**				0.616 (0.735)
Extroverted	68 (38.0)	26 (38.2)	42 (37.8)	
Introverted	111 (62.0)	42 (61.8)	69 (62.2)	
**Range of burns (%)**	19.31 ± 17.17	16.7 ± 14.63	20.9 ± 18.44	−1.678 (0.095)
**Pain caused by burns**	4.68 ± 2.44	4.40 ± 2.28	4.85 ± 2.53	−1.199 (0.232)
**Number of burn sites**				2.786 (0.426)
1	81 (45.3)	28 (41.2)	53 (47.7)	
2	53 (29.6)	23 (33.8)	30 (27.0)	
3	35 (19.6)	15 (22.1)	20 (18.0)	
≥ 4	10 (5.5)	2 (2.9)	8 (7.3)	
**Difficulty in daily life due to burns**				1.250 (0.535)
None	7 (3.9)	3 (4.4)	4 (3.6)	
A little	73 (40.8)	31 (45.6)	42 (37.8)	
A lot	99 (55.3)	34 (50.0)	65 (58.6)	

Note.

^†^ *SD*, standard deviation

### 4.2 Mean score difference by treatment phase

The mean score differences among the factors related to patients with severe burns by treatment phase are shown in Table 2. Depressive symptoms had a statistically significant mean score difference between the two phases (*t* = −2.681, *p* = .008). Among the subcategories of posttraumatic growth, changes in self‐perception had a statistically significant mean score difference between the two phases (*t* = 2.812, *p* = .006).

**Table 2:** Mean Score Difference by Treatment Phase (*N* = 179)

Variables	Acute phase (*N* = 68, 38%)	Rehabilitation phase (*N* = 111, 62%)	*t* (*p*)
	Mean ± *SD*†	Mean ± *SD*	
Depressive symptoms	15.93 ± 9.68	20.55 ± 13.31	−2.681 (0.008)
Social support	83.56 ± 22.75	85.10 ± 20.19	−0.473 (0.637)
Emotional support	3.37 ± 0.94	3.40 ± 0.80	−0.186 (0.853)
Evaluative support	3.42 ± 0.87	3.49 ± 0.81	−0.549 (0.584)
Informational support	3.29 ± 0.98	3.39 ± 0.87	−0.691 (0.491)
Material support	3.28 ± 0.94	3.34 ± 0.85	−0.443 (0.659)
Posttraumatic growth	44.13 ± 15.01	40.32 ± 15.71	1.601 (0.111)
Changes in interpersonal relationships	2.93 ± 1.08	2.65 ± 1.03	1.728 (0.086)
Changes in self‐perception	3.01 ± 0.99	2.54 ± 1.21	2.812 (0.006)
Discovering new possibilities	2.51 ± 1.15	2.72 ± 1.08	−1.239 (0.217)
Increased spiritual and religious interest	1.96 ± 1.46	1.84 ± 1.34	0.574 (0.567)

Note

^†^ *SD*, Standard Deviation

### 4.3 Correlations between posttraumatic growth and other variables by treatment phase

Table 3 presents the results of the analysis of the relationship between posttraumatic growth and other variables by treatment phase. Posttraumatic growth and depressive symptoms showed a statistically significant negative relationship for patients in both the acute phase (r = −0.257, *p* = .035) and the rehabilitation phase (r = −0.378, *p* < .001). Posttraumatic growth and social support showed a statistically significant positive relationship for patients in both the acute phase (r = 0.401, *p* = .001) and the rehabilitation phase (r = 0.510, *p* < .001).

**Table 3:** Correlations between Posttraumatic Growth and Other Variables by Treatment Phase (*N* = 179)

Variables	Posttraumatic growth	
	Acute phase (*N* = 68) *r* (*p*)	Rehabilitation phase (*N* = 111) *r* (*p*)
Depressive symptoms	−0.257 (0.035)	−0.378 (< 0.001)
Social support	0.401 (0.001)	0.510 (< 0.001)

### 4.4 Factors associated with posttraumatic growth by treatment phase

Multicollinearity, residuals and singular values were identified to verify the assumptions of the regression analysis for the independent variables. First, after identifying multicollinearity among the independent variables, it was identified that the predictor variables were independent as there were no explanatory variables with a correlation higher than 0.800 and there were no problems of auto‐correlation as the Durbin–Watson statistic was 1.848 for the acute phase and 1.606 for the rehabilitation phase. The tolerance was between 0.836 and 0.863, which is less than 1.0. In addition, there was no problem of multicollinearity as the variance inflation factor was between 1.158 and 1.196, not exceeding 10, which is the threshold. The results for testing the residual assumption showed that the data satisfied the assumptions of linearity, normality of errors and homoscedasticity and the maximum value of Cook's distance for identifying any singular value did not exceed 1.0:0.314 for the acute phase and 0.090 for the rehabilitation phase. Therefore, as all the assumptions were satisfied, it was determined that the results of the regression analysis were reliable.

On investigating the factors associated with posttraumatic growth in patients with severe burns in the acute and rehabilitation phases (Table 4), social support was found to be statistically significant in the acute phase (*β* = 0.355, *t* = 2.929, *p* = .005), explaining 14.9% of the variance (*F* = 6.884, *p* = .002). Depressive symptoms (*β* = −0.205, *t* = −2.321, *p* = .022) and social support (*β* = 0.427, *t* = 4.827, *p* < .001) were associated with posttraumatic growth in the rehabilitation phase and explained 28.2% of the variance (*F* = 22.584, *p* < .001).

**Table 4:** Factors Associated with Posttraumatic Growth by Treatment Phase (*N* = 179)

Variables	Posttraumatic growth			
	Acute phase (*N* = 68)		Rehabilitation phase (*N* = 111)	
	*β*	*t* (*p*)	*β*	*t* (*p*)
Depressive symptoms	−0.125	−1. 034 (0.305)	−0.205	−2.321 (0.022)
Social support	0.355	2.929 (0.005)	0.427	4.827 (< 0.001)
Adjusted *R^2^*		0.149		0.282
*F* (*p*)		6.884 (0.002)		22.584 (< 0.001)

## 5 DISCUSSION

This study examined differences in the level of depressive symptoms, social support and posttraumatic growth among patients with severe burns by treatment phase. Participants were categorized into the acute and rehabilitation phases and factors associated with posttraumatic growth were identified to provide basic data for the development of clinical nursing interventions focusing on the psychosocial aspects of severe burns.

There was a statistically significant difference in the level of depressive symptoms between the two groups: patients in the acute phase had mild depression with an average score of 15.93 whereas those in the rehabilitation phase had moderate depression with an average score of 20.55. This result is not congruent with Park’s (2016) finding that patients with burn injuries who needed to be hospitalized had severe depression. The difference may be owing to the fact that Park’s (2016) study did not account for the number of days since the burn, severity of the burn and treatment phase when explaining the level of depression. Thus, there is a need for repeated studies for considering the factors relevant to each treatment phase. The first year after the burn incident is particularly important for patients with severe burns because that is when they are most susceptible to psychological pain or fear related to having to go through outpatient treatment and surgery even after discharge. Their depression levels tend to increase from the secondary stress of having to adapt to everyday life, memory of the incident and financial hardships (Son, 2005). Hence, clinical nurses should accurately identify patients’ psychological states by treatment phase and accordingly provide appropriate care.

The two groups did not have a statistically significant difference in terms of social support levels; however, patients in the rehabilitation phase had higher social support than did those in the acute phase in most subcategories. The above results may be owing to the fact that patients with burn injuries in the acute phase underwent longer hospitalization and treatment.

The two groups did not have a statistically significant difference in terms of overall posttraumatic growth; however, a statistically significant difference was observed in the changes in self‐perception. The subcategory of self‐perception asked if the patient overcame difficulties and became more receptive, positive and mentally stronger, which has a context similar to that of depression in participants in both the acute and rehabilitation phases. Specifically, patients in the acute phase who have received urgent treatment for physical injuries enter the recovery stage, thus becoming more appreciative of life and determined to overcome difficulties (Weon & Han, 2018). However, those in the rehabilitation phase, after undergoing the acute phase, encounter limitations through repeated treatments and feel disappointed and discouraged, thus ultimately developing negative self‐perceptions (Lee, 2017). Therefore, patients in the rehabilitation phase need nursing interventions where they can participate in self‐help groups, improve positive self‐perceptions and receive supportive communication from nurses.

A statistically significant inverse correlation between depressive symptoms and posttraumatic growth was observed among patients in both the acute and rehabilitation phases. A study by Park (2011) with 241 young adults examined the relationship between traumatic incidents and depression and reported an inverse correlation of the total score of depressive symptoms with posttraumatic growth and all subcategories, congruent with this study's findings. Conversely, social support had a positive correlation with posttraumatic growth in this study. Previous studies (Han & Lee, 2011; Park, Jeong, Lee, & Kang, 2017) have revealed that individuals who have been injured are more likely to experience positive changes if they receive more support from others, consistent with the results of this study.

Social support was the common factor for both groups. Patients with burns on 80% or more of the total body surface area in the acute phase have a higher survival rate if they have high social support (Muangman et al., 2005). Patients with breast cancer who experience physical changes as patients with burns do are more likely to have attachment to life and undergo posttraumatic growth if they have supportive and positive relationships with their loved ones (Lelorain, Bonnaud‐Antignac, & Florin, 2010), which also corresponds to the findings of this study. Internal factors such as an individual's desire to find strength and external factors such as social support influence patients’ posttraumatic growth such that they become more positive, begin to understand others’ pain and gain the confidence to overcome difficulties (Song et al., 2009). With a higher level of social support, patients with burns experience reduced physical pain and recovery times through accelerated rehabilitation (Davidson, Bowden, Tholen, James, & Feller, 1981; Lee, 2010); hence, social support is a necessary external factor for patients in the rehabilitation phase. Meanwhile, the severity of depression was associated with posttraumatic growth among patients in the rehabilitation phase. A seven‐year follow‐up of breast cancer survivors revealed similar results, as did Carver and Antoni’s (2004) study, which reported that individuals with high posttraumatic growth had a low level of depressive symptoms.

Psychological growth stems from one's desire to find meaning in the traumatic event and these cognitive approaches can lead to positive changes in psychological states (Martin et al., 2017). Patients with major burns must develop a cognitive understanding of the occurrences after the injury and these contemplative processes may lead to posttraumatic growth. Moreover, clinical nurses who provide care for patients with severe burns must provide integrative care by establishing a support system where self‐help groups are readily available to offer psychological support in addition to treatment support so that the patients can develop adequate self‐understanding and self‐respect.

### 5.1 Limitations

Despite this study's valuable findings, it has some limitations that need to be addressed. First, as this study was a cross‐sectional study, it could not make causal inferences. Second, as this study was conducted in a single hospital, further studies with larger samples are required to examine the factors apart from depression and social support that influence posttraumatic growth.

### 5.2 Implications for clinical practice

In our study, we elucidate that patients with severe burns in the rehabilitation phase of treatment have higher levels of depressive symptoms than those in the acute phase and that depressive symptoms affect posttraumatic growth significantly only for patients in the rehabilitation phase. This can act as an important insight that enables healthcare professionals to recognize that severe burn patients need sustained psychological support and management of depressive symptoms, not only in the acute phase, but also in the rehabilitation phase. In both the groups, social support was a common factor affecting posttraumatic growth in severe trauma patients with burns. Thus, building the patient's willingness to live and ensuring meaningful sources of social support with the help of clinical interventions may contribute to the patient's recovery through positive psychological changes such as posttraumatic growth.

## 6 CONCLUSIONS

In this study, for posttraumatic growth, social support was important in the acute phase group, whereas both fewer depressive symptoms and social support were important in the rehabilitation phase group. In terms of severity, the acute phase group had mild depression. However, the rehabilitation phase group had moderate depression and a low level of positive self‐perception. These results signify that each treatment phase needs a differentiated approach during psychosocial interventions. Therefore, we suggest that counselling intervention and support be extended to patients with severe burns. In the early stages of the crisis of severe burns, the diagnosis of and intervention in depressive symptoms are performed in the clinical domain. However, as can be seen from the results of this study, although depressive symptoms may be more serious in the rehabilitation process, healthcare workers tend to overlook them. Therefore, in the future, healthcare workers should establish a system wherein depressive symptoms in patients with severe burns are not neglected and they can benefit from psychosocial interventions while living in the community. Furthermore, nurses should apply an integrated approach of psychological and nursing support, so that patients’ posttraumatic growth is enhanced based on appropriate self‐understanding and self‐respect.

## CONFLICT OF INTEREST

None.
